# Effects of Using a Digital Peer-Supported App on Glycated Hemoglobin Changes Among Patients With Type 2 Diabetes: Prospective Single-Arm Pilot Study

**DOI:** 10.2196/72659

**Published:** 2025-05-20

**Authors:** Shota Yoshihara, Kayoko Takahashi, Hiroyuki Kawaguchi, Nozomi Harai, Kyoichiro Tsuchiya

**Affiliations:** 1 Department of Rehabilitation Sciences, Kitasato University Graduate School of Medical Sciences, 1-15-1 Kitasato, Minami-ku, Sagamihara, Kanagawa, 252-0373, Japan, 81 042-788-9694; 2 A10 Lab Inc, Tokyo, Japan; 3 Department of Occupational Therapy, School of Allied Health Science, Kitasato University, Kanagawa, Japan; 4 Department of Diabetes and Endocrinology, University of Yamanashi Hospital, Yamanashi, Japan

**Keywords:** diabetes type 2, T2DM, self-management, digital health, HbA1c, lifestyle intervention, digital intervention, type 2 diabetes mellitus, glycated hemoglobin

## Abstract

**Background:**

Controlling glycated hemoglobin (HbA_1c_) levels can be challenging for patients with type 2 diabetes mellitus (T2DM). Peer support promotes HbA_1c_ control, and a digital peer-supported app designed for group interactions may enable patients with T2DM to encourage one another to achieve better HbA_1c_ outcomes. However, no studies have investigated the use of digital peer-supported apps to control HbA_1c_ levels in patients with T2DM.

**Objective:**

This pilot study aimed to explore the effects of a digital peer-supported app on HbA_1c_ control in patients with T2DM.

**Methods:**

This prospective single-arm pilot study enrolled patients with T2DM who owned smartphones and visited medical institutions in Japan. During the 3-month intervention, participants used a digital peer-supported app in addition to receiving standard care. This app allowed participants to share activity logs and concerns via a chat function to improve HbA_1c_ levels through mutual engagement and encouragement. The primary outcome was the change in HbA_1c_ levels, measured at health care facilities at baseline and after 3 months. The secondary outcomes were body weight and blood pressure, with the most recent data obtained from hospitals and clinics. Physical activity (≥1 hour/day) was assessed at the same time points using a self-reported questionnaire.

**Results:**

The study included 21 participants with a median age of 56 (IQR 51‐61) years, of which 13 (61.9%) were female. After using the digital peer-supported app for 3 months, the participants’ HbA_1c_ levels significantly decreased from 7.1% (SD 0.6%) at baseline to 6.9% (SD 0.1%) (*P=*.04). Similarly, participants’ body weight decreased from 70.7 (SD 12.7) kg to 69.9 (SD 12.4) kg (*P* =.004) through app use. Although blood pressure decreased slightly from 128.2 (SD 12.5) mm Hg to 126.0 (SD 12.9) mm Hg, this change was not statistically significant (*P*=.20). Additionally, the proportion of participants engaged in ≥1 hour of daily physical activity significantly increased from 23.5% (n=4) to 58.5% (n=10) (*P=*.03).

**Conclusions:**

In addition to receiving standard clinical care, the use of a digital peer-supported app may significantly lower HbA_1c_ levels in patients with T2DM by promoting healthy behaviors.

## Introduction

Type 2 diabetes mellitus (T2DM) has become a global public health issue that significantly affects 90% of the 537 million patients with T2DM worldwide [1]. T2DM and its complications significantly impact patients and the society by increasing health care costs and reducing life expectancy and quality [2]. Glycated hemoglobin (HbA_1c_) is the gold standard for measuring average blood glucose levels over 3 months and predicting the relative risk of diabetes complications [3,4]. A previous study revealed that adopting healthy behaviors (ie, maintaining a healthy body weight, following a healthy diet, exercising, avoiding smoking, and avoiding drinking alcohol) is an effective strategy for controlling HbA_1c_ levels in patients with T2DM [5]. However, adopting and maintaining healthy behaviors independently can be challenging for patients with T2DM [6].

Peer support, defined as mutual assistance among individuals with shared experiences or challenges, may be an effective strategy for enhancing healthy behaviors in patients with T2DM [7]. A meta-analysis of 13 randomized controlled trials that focused on in-person peer support among patients with diabetes has indicated that patients receiving peer support experienced a significant reduction in HbA_1c_ levels of 0.57% (95% CI 0.36‐0.78) on average, compared to those without peer support, across interventions durations ranging from 3 to 24 months [8]. However, traditional lifestyle counseling such as peer support in clinical settings, which relies heavily on face-to-face interactions and significant human and time resources, provides limited peer support for patients with T2DM that is often restricted to infrequent hospital visits [9].

With advancements in digital health technology, a digital peer-supported app may provide new opportunities for patients with T2DM to connect and support each other in virtual spaces, thus enabling those with T2DM to collaborate toward the goal of controlling HbA_1c_ levels. Using digital peer-supported apps, known as group-type apps, enable individuals to encourage each other to engage in healthy behaviors [10-12]. However, the effectiveness of such digital peer support interventions in T2DM management remains largely unclear.

This pilot study aimed to explore the effects of a digital, peer-supported app on HbA_1c_ control in patients with T2DM.

## Methods

### Study Design

This 3-month single-arm intervention study used a pre–post evaluation design. According to the transtheoretical model [13], a 3-month period is widely regarded in many studies as sufficient for individuals to progress from the “preparation stage” to the “action stage” and, subsequently, to the “maintenance stage.” In addition, the 3-month period is both physiologically justified and conceptually appropriate for evaluating behavioral and metabolic changes [3,4], as HbA_1c_ reflects the average blood glucose levels over the preceding 2‐3 months, which corresponds to the approximately 120-day lifespan of red blood cells [14].

This study was conducted between December 2021 and June 2022 as an industry-government-academia collaboration among local governments, app-making companies, and universities. It was part of the “TRY! YAMANASHI! Pilot Experiment Support Project (FY2021, First Term)” in Yamanashi Prefecture. The program supports the social implementation of advanced technologies and services developed by startups and other innovators to address regional issues and create public value. Support also offered multifaceted assistance (eg, funding, expert consultation, partnerships with local resources, and public outreach) for projects that had advanced to the practical application stage through prior pilot testing.

### Ethical Considerations

Informed consent was obtained from all participants before their enrollment. This study adhered to the ethical principles outlined in the Declaration of Helsinki and was approved by the independent Ethics Review Committee of Healthcare Systems Co., Ltd. (Approval No. 2130). Participants received a financial reward of 5000 JPY (US $34.70) in the form of a gift card when the end-of-study data were delivered (ie, HbA_1c_ levels at both time points and patient-reported outcomes at baseline and at 3 months). All collected data were anonymized prior to analysis to ensure participant confidentiality and privacy.

### Participant Recruitment

Participants were recruited through convenience sampling and began using the digital peer-supported app upon recruitment. An explanation of the study and its objectives was provided by physicians when they visited the clinic and hospital. The referral process involved the following scenarios: (1) during a health consultation at a hospital; (2) as part of a diabetes program at a hospital; and (3) during a medical consultation at a clinic.

The inclusion criteria were (1) individuals who had been fully informed about the objectives and details of the study, possessed the capacity to consent, had understood the information thoroughly, and have freely agree to participate by providing written consent; (2) Japanese men and women aged 20-74 years at the time of consent; (3) patients with T2DM or prediabetes with an HbA_1c_ level of ≥6.0% and <8.5%; and (4) individuals who owned and could operate a smartphone (iOS or Android) capable of installing the app. The exclusion criteria were (1) diseases that could interfere with the study, as determined by the medical institution conducting the study, and (2) severe symptoms or disabilities due to lifestyle-related diseases, as determined by the medical institution.

### Intervention Program

**Figure 1.**
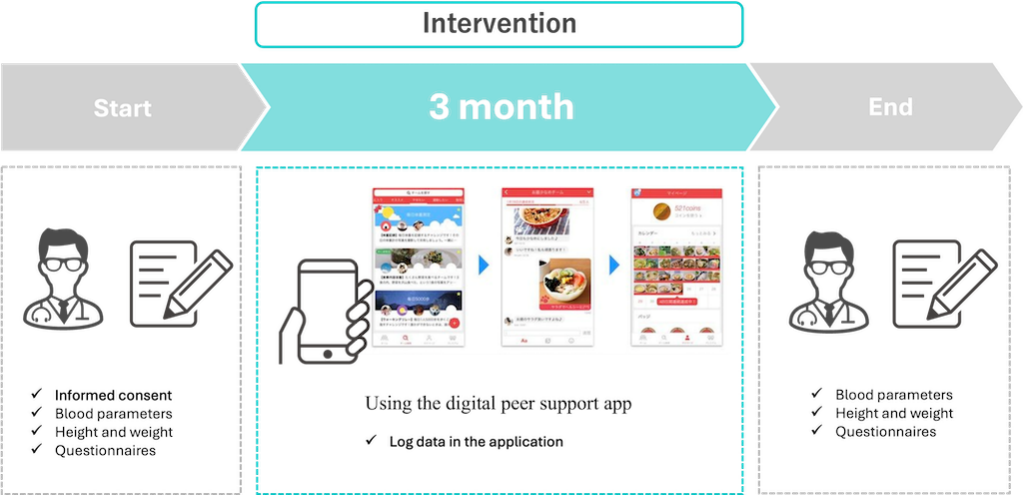
Experimental flow of this study.

The experimental setup is shown in Figure 1. The participants began using the digital peer-supported app after being introduced to the app by physicians and downloading it. During the initial login, they received tutorials within the app. Participants were encouraged to join diabetes management teams within the digital peer-supported app and use this app for 3 months while continuing their treatment according to the standards of care, including any necessary adjustments to their diabetes pharmacotherapy. They were instructed to complete questionnaires at the beginning and end of the program to evaluate changes in their lifestyle habits and health behaviors. These questionnaires were designed to confirm participant sex, age, and health behaviors. HbA_1c_ levels, blood pressure, and body weight were measured at the beginning and end of the study, using the most recent data from hospitals and clinics.

### Digital Peer-Supported App

**Figure 2.**
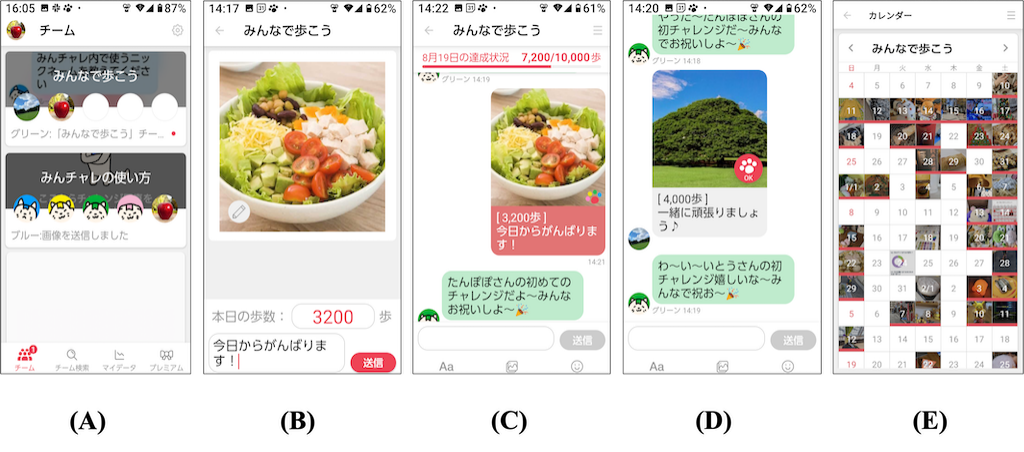
Examples of app screens. (A) Select a group. (B) Post a photo taken that day and comment on the day’s events to the group. (C) The contents of the postings are displayed in the group. (D) Respond to posts by group members. (**E**) Check the records.

The study used “Minchalle,” a commercially available digital peer-supported app [11]. This digital peer-supported app was developed by A10 Lab Inc. in June 2015 with an initial release in November 2015. It is available for both Android and iPhone devices. The digital peer-supported app created group chats of up to five participants that aimed at controlling their HbA_1c_ levels, and participants were anonymously assigned to each group (Figure 2). Once daily, the participants posted a set of daily activities to control their HbA_1c_ levels, along with photos and comments in a group chat box. The main functions of the digital peer-supported app used in this study were (1) posting photos, step counts, and comments about the day; (2) posting approvals from group members; (3) setting step-count goals on a group basis; and (4) providing feedback from assistant robots. Participants could post comments or photos more than once a day and enjoy interacting with other members; however, daily participation was not obligatory, and the participants were allowed to freely use the app. Examples of the user interfaces of digital peer-supported apps are provided in the Multimedia Appendix 1.

### Measurements

We collected basic demographic information at baseline, including sex (male or female), age (in continuous years), alcohol consumption (categorized as nondrinker, former drinker, occasional drinker, or nearly daily drinker), and smoking status (categorized as nonsmoker, former smoker, or current smoker).

The primary outcome of this study was the difference in HbA_1c_ levels, which were evaluated using values measured at health care facilities immediately during the participants’ visits at baseline and at the 3-month follow up. All participants’ baseline and 3-month follow-up values were measured at the same health care facilities using the same reagents. The secondary outcomes included differences in body weight and blood pressure, which were also obtained from the health care facilities. At the same time points, physical activity (≥1 hour/day) was evaluated using a self-reported questionnaire: *“*Do you engage in walking or equivalent physical activity for >1 hour per day in your daily life?*”* Responses were recorded as either “yes” or “no.”

### Statistical Analysis

We performed the Wilcoxon signed-rank test to assess the overall significance of differences in HbA_1c_ levels. Similarly, changes in secondary outcomes such as blood pressure and body weight were evaluated using the Wilcoxon signed-rank test to compare baseline and 3-month follow-up values. Additionally, McNemar test was used to analyze paired binary data, specifically to compare baseline and 3-month follow up values for physical activity, ≥1 hour/day (%).

All analyses were performed using the Stata software (version 18.0; StataCorp LLC). Statistical significance was set at *P*<.05 (2-tailed).

## Results

**Figure 3.**
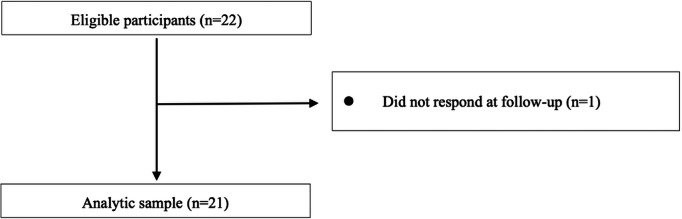
Flow diagram of study participant recruitment (glycated hemoglobin, blood pressure, and body weight).

**Figure 4.**
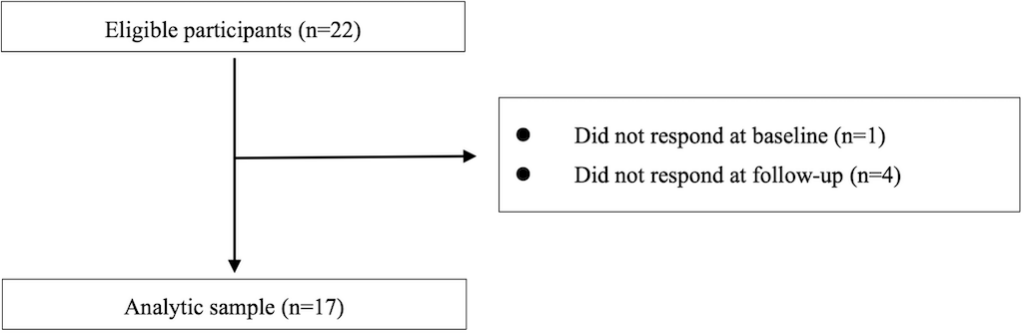
Flow diagram of study participant recruitment (physical activity ≥1 hour/day).

The eligibility assessment and enrolment processes are shown in Figures3  4, respectively. Based on HbA_1c_ levels, blood pressure, and weight (Figure 3), 22 participants were initially enrolled, and app usage was confirmed using the company database. One participant was excluded because of nonresponsiveness at follow-up, leaving 21 participants (95.5%) with complete HbA_1c_ levels, blood pressure, and weight data for analysis. Similarly, based on questionnaire data for physical activity ≥1 hour/day (%) (Figure 4), 22 participants were enrolled, and app usage was confirmed.

Table 1 shows the baseline characteristics of the 21 participants, including 3 (14.3%) individuals with prediabetes. The median participant age was 56 (IQR 51‐61) years, and 61.9% (n=13) were females. Among the patients, 52.4% (n=11) were nonsmokers, 23.8% (n=5) were former smokers, and 23.8% (n=5) were current smokers. Regarding alcohol intake, 28.6% (n=6) of the study cohort were nondrinkers, 4.8% (n=1) was a former drinker, 61.9% (n=13) were occasional drinkers, and 4.8% (n=1) was a daily drinker. At baseline, 23.5% (n=4) of the participants reported ≥1 hour/day of physical activity. The median HbA_1c_, blood pressure, and body weight were 7.1% (IQR 6.7%‐7.3%), 127 (IQR 121‐135) mm Hg, and 70.4 (IQR 61.9‐80.4) kg, respectively.

**Table 1.** Baseline participant characteristics.

Baseline characteristics	Participants^a^ (N=21)
Sex, n (%)	
Male	8 (38.1)
Female	13 (61.9)
Age (year), median (IQR)	56 (51-61)
Smoking status, n (%)	
Nonsmoker	11 (52.4)
Former smoker	5 (23.8)
Current smoker	5 (23.8)
Alcohol intake, n (%)	
Nondrinker	6 (28.6)
Former drinker	1 (4.8)
Occasional drinker	13 (61.9)
Daily drinker	1 (4.8)
Physical activity ≥1 hour/day (n=17)^b^, n (%)	4 (23.5)
HbA_1c_^c^ (%), median (IQR)	7.1 (6.7-7.3)
Blood pressure (mm Hg), median (IQR)	127 (121-135)
Body weight (kg), median (IQR)	70.4 (61.9-80.4)

a Data are shown as medians and quartiles for continuous variables and numbers (percentages) for categorical variables.

b Data were available only for 17 participants.

c HbA_1c_: glycated hemoglobin.

Table 2 presents the results from the Wilcoxon signed-rank test, showing changes in HbA_1c_ levels from baseline to the 3-month follow-up. The mean baseline HbA_1c_ value for the intraindividual control group was 7.1% (SD 0.6%), which decreased by an average of 0.1% (SD 0.3%) to reach a 3-month follow-up value of 6.9% (SD 0.1%) (*P*=.04). As an additional metabolic parameter, patients reported their weight before and after 3 months. The groups’ mean baseline blood pressure was recorded at 128.2 (SD 12.5) mm Hg and decreased on average by 2.1 (SD 9.4) mm Hg, resulting in a follow-up value of 126.0 (SD 12.9) mm Hg. However, this change in blood pressure was not statistically significant (*P*=.20). In addition, the mean baseline weight for the intraindividual control group was 70.7 (SD 12.7) kg, which decreased on average by 0.8 (SD 1.0) kg, resulting in a 3-month follow-up value of 69.9 (SD 12.4) kg (*P*.004). Finally, the proportion of participants engaging in ≥1 hour of daily physical activity significantly increased from 23.5% (n=4) to 58.5% (n=10) (*P=*.03).

**Table 2.** Changes in glycemic control, metabolic parameters, and physical activity.

Parameters	Baseline values (n=21)	Follow-up values (at 3 months)	Change	*P* value
HbA_1c_ (%), mean (SD)	7.1(0.6)	6.9 (0.1)	−0.1 (0.3)	.04^a^
Blood pressure (mm Hg), mean (SD)	128.2 (12.5)	126.0 (12.9)	−2.1 (9.4)	.20^a^
Body weight (kg), mean (SD)	70.7 (12.7)	69.9 (12.4)	−0.8 (1.0)	<.004^a^
Physical activity ≥1 hour/day (n=17), n (%)	4 (23.5)	10 (58.5)	—^b^	.03^c^

a Wilcoxon signed-rank test.

b Not applicable.

c McNemar test.

## Discussion

### Principal Findings

Our findings showed that a 3-month intervention using a digital peer-supported app led to a modest but statistically significant reduction in HbA_1c_ levels among patients with T2DM, indicating improved glycemic control. Additionally, self-reported frequency of physical activity improved significantly, highlighting the potential of this app to promote healthier behaviors. The findings of this study suggest that digital, peer-supported apps may serve as promising practical tools for the management of T2DM.

Moreover, the intervention using a digital peer-supported app significantly reduced HbA_1c_ levels among patients with T2DM, which is consistent with findings from previous studies that used nongroup-based, single-user apps for blood glucose control [15-17]. Peer support, grounded in the social cognitive theory, could effectively aid in blood glucose control when implemented through a digital peer-supported app [18].

Our results may be attributed to specific app design features, including reminder prompts and chat functions. One possible explanation is that communication via chats may also impact individual decision-making and behavior, shaping online community norms to maintain healthy behaviors [19]. These community norms may offer promising ways to improve adherence to diet and exercise recommendations by providing real-time reminders and emotional support from peers [20].

Previous studies on diabetes apps have highlighted the importance of chat-based communication in promoting healthy behavioral change, particularly by reinforcing positive actions such as initiating exercise, which can be more effective than restrictive measures [21]. Alternatively, real-time chats and reminders may help users remain frequently aware of their goals and consistently track their behaviors, which may facilitate effective acquisition, learning, and app of health information [15,22]. Although digital peer support platforms carry the risk of misinformation or inappropriate advice, no such incidents were reported during the study period. This may be attributed to the implementation of a safety-monitoring system, which included content oversight by the app provider and a dedicated research contact point. Future interventions should incorporate risk-management protocols to ensure safe and effective peer communication.

Our results showed a statistically significant change in HbA_1c_ levels, although the reduction did not reach a threshold considered meaningful in a clinical context [23]. One possible explanation for this finding is that the participants may have had low baseline HbA_1c_ levels. In a recent meta-analysis of online medicine, higher baseline HbA_1c_ levels (>7.5% or >8.0%), younger age (<55 years), and shorter duration of diabetes (<8.5 years and <7 years) were found to be associated with increased benefits [23]. Our study participants had lower baseline HbA_1c_ levels than those in other studies [15,16,24]. Future studies should aim to identify demographic or behavioral subgroups more likely to achieve HbA_1c_ reduction using digital peer-supported apps.

### Strengths and Limitations

This study reported that a 3-month intervention using a digital peer-supported app led to a modest but statistically significant reduction in HbA_1c_ levels, which indicates improved glycemic control among patients with T2DM. Additionally, self-reported frequency of physical activity improved significantly, highlighting the potential of this app in promoting healthier behaviors. By minimizing interference from study personnel and addressing only technical aspects, the app was evaluated under real-life conditions to generate realistic and practical evidence. Therefore, digital peer-supported apps may offer cost-effective and scalable tools for managing diabetes and supporting lifestyle changes.

However, this study had several limitations that should be noted when interpreting our findings. First, as this was a single-arm interventional study with a small sample size, the analysis relied on classical statistical methods and a pre-post comparison, which makes it difficult to exclude the influence of confounding factors, such as baseline HbA_1c_, age, or sex, through statistical adjustment. Although the reductions in HbA_1c_ and body weight were statistically significant, their relatively small magnitude along with the absence of detailed data on app usage, user engagement, and acceptability limit our ability to attribute these improvements solely to digital peer support interventions. These improvements may also have been mediated by increased adherence to treatment rather than by direct effects. To enhance the validity and generalizability of these findings, a multicenter randomized controlled trial with a larger and more diverse sample size and a parallel control group is currently underway (UMIN000056609). Second, this study used only HbA_1c_ level as the primary outcome measure. Although HbA_1c_ is a reliable indicator of long-term glycemic control and reflects average blood glucose levels over the past 2‐3 months, it is subject to a time lag between behavioral changes and measurable outcomes. In contrast, continuous glucose monitoring devices can capture interstitial glucose levels in near real time, allowing immediate feedback and short-term trend analysis [25]. Future studies may benefit from combining long-term markers such as HbA_1c_ with continuous glucose monitoring to investigate the effects of interventions more comprehensively. Third, unmeasured factors such as medication adjustments and dietary habits during the study period may have influenced changes in HbA_1c_ levels. Although the duration since the diagnosis of diabetes may influence a participants’ engagement with peer support and intervention outcomes, this information was not collected in the present study and should be considered in future studies. Fourth, this study did not collect information on personal or behavioral traits. As previous studies have shown that the stage of behavioral change may influence continued app usage [26], future studies should examine how individual characteristics affect long-term engagement in digital peer support. Finally, the sample size of 21 participants was relatively small and may have been insufficient to adequately detect significant changes in health outcomes. A formal sample size calculation was not conducted due to the lack of prior studies on the effectiveness of digital peer support on health outcomes. Future studies should assess the impact of digital peer-supported apps on HbA_1c_ levels, enabling appropriate calculation of effect sizes.

### Conclusions

The use of a digital peer-supported app intervention may significantly lower HbA_1c_ levels in patients with T2DM after 3 months. In T2DM populations capable of using the app, this intervention could promote healthier behaviors, in addition to regular clinical care, and may help reduce HbA_1c_ levels.

## Supplementary material

10.2196/72659 Multimedia Appendix 1 Examples of the user interfaces of digital peer-supported apps are provided.

## Abbreviations

HbA_1c_: glycated hemoglobin
T2DM: type 2 diabetes mellitus

## References

[1] Sun H, Saeedi P, Karuranga S (2022). IDF Diabetes Atlas: global, regional and country-level diabetes prevalence estimates for 2021 and projections for 2045. Diabetes Res Clin Pract.

[2] Association American Diabetes (2018). Updates to the Standards of Medical Care in Diabetes-2018. Diabetes Care.

[3] Nathan DM, Genuth S, Diabetes Control and Complications Trial Research Group (1993). The effect of intensive treatment of diabetes on the development and progression of long-term complications in insulin-dependent diabetes mellitus. N Engl J Med.

[4] Stratton IM, Adler AI, Neil HA (2000). Association of glycaemia with macrovascular and microvascular complications of type 2 diabetes (UKPDS 35): prospective observational study. BMJ.

[5] Davies MJ, D’Alessio DA, Fradkin J (2018). Management of hyperglycemia in type 2 diabetes, 2018. a consensus report by the American Diabetes Association (ADA) and the European Association for the Study of Diabetes (EASD). Diabetes Care.

[6] Ried-Larsen M, MacDonald CS, Johansen MY (2018). Why prescribe exercise as therapy in type 2 diabetes? We have a pill for that!. Diabetes Metab Res Rev.

[7] Riessman F (1990). Restructuring help: a human services paradigm for the 1990s. American J Comm Psychol.

[8] Qi L, Liu Q, Qi X, Wu N, Tang W, Xiong H (2015). Effectiveness of peer support for improving glycaemic control in patients with type 2 diabetes: a meta-analysis of randomized controlled trials. BMC Public Health.

[9] Ricci-Cabello I, Ruiz-Pérez I, Rojas-García A, Pastor G, Rodríguez-Barranco M, Gonçalves DC (2014). Characteristics and effectiveness of diabetes self-management educational programs targeted to racial/ethnic minority groups: a systematic review, meta-analysis and meta-regression. BMC Endocr Disord.

[10] Okamoto M, Saito Y, Nakamura S (2024). Smartphone-based digital peer support for a walking intervention among public officers in Kanagawa prefecture: single-arm pre- and postintervention evaluation. JMIR Form Res.

[11] Tabira K, Oguma Y, Yoshihara S (2024). Digital peer-supported app intervention to promote physical activity among community-dwelling older adults: nonrandomized controlled trial. JMIR Aging.

[12] Takebayashi M, Namba M, Koyama T (2024). Impact on step count by commitment-based health application. PLoS One.

[13] Prochaska JO, Velicer WF (1997). The transtheoretical model of health behavior change. Am J Health Promot.

[14] Sherwani SI, Khan HA, Ekhzaimy A, Masood A, Sakharkar MK (2016). Significance of HbA1c test in diagnosis and prognosis of diabetic patients. Biomark Insights.

[15] Bretschneider MP, Klásek J, Karbanová M, Timpel P, Herrmann S, Schwarz PEH (2022). Impact of a digital lifestyle intervention on diabetes self-management: a pilot study. Nutrients.

[16] Bretschneider MP, Roth L, Schwarz PEH (2023). Effectiveness of a digital health application for the treatment of diabetes type II-a pilot study. J Clin Med.

[17] Waki K, Fujita H, Uchimura Y (2014). DialBetics: a novel smartphone-based self-management support system for type 2 diabetes patients. J Diabetes Sci Technol.

[18] Ginis KAM, Nigg CR, Smith AL (2013). Peer-delivered physical activity interventions: an overlooked opportunity for physical activity promotion. Transl Behav Med.

[19] Chancellor S, Hu A, De Choudhury M Norms matter: contrasting social support around behavior change in online weight loss communities.

[20] Saffari M, Ghanizadeh G, Koenig HG (2014). Health education via mobile text messaging for glycemic control in adults with type 2 diabetes: a systematic review and meta-analysis. Prim Care Diabetes.

[21] Zhang Y, Li X, Luo S (2019). Use, Perspectives, and attitudes regarding diabetes management mobile apps among diabetes patients and diabetologists in China: national web-based survey. JMIR Mhealth Uhealth.

[22] Lim SL, Tay MHJ, Ong KW (2022). Association between mobile health app engagement and weight loss and glycemic control in adults with type 2 diabetes and prediabetes (D’LITE Study): prospective cohort study. JMIR Diabetes.

[23] Timpel P, Oswald S, Schwarz PEH, Harst L (2020). Mapping the evidence on the effectiveness of telemedicine interventions in diabetes, dyslipidemia, and hypertension: an umbrella review of systematic reviews and meta-analyses. J Med Internet Res.

[24] Pamungkas RA, Usman AM, Chamroonsawasdi K (2022). A smartphone application of diabetes coaching intervention to prevent the onset of complications and to improve diabetes self-management: a randomized control trial. Diabetes Metab Syndr.

[25] Battelino T, Alexander CM, Amiel SA (2023). Continuous glucose monitoring and metrics for clinical trials: an international consensus statement. Lancet Diabetes Endocrinol.

[26] Tong HL, Laranjo L (2018). The use of social features in mobile health interventions to promote physical activity: a systematic review. NPJ Digit Med.

[27] (2025). GPT-4. OpenAI.

[28] (2025). Grammarly.

